# A dsRNA-binding protein of a complex invertebrate DNA virus suppresses the *Drosophila* RNAi response

**DOI:** 10.1093/nar/gku910

**Published:** 2014-10-01

**Authors:** Alfred W. Bronkhorst, Koen W.R. van Cleef, Hanka Venselaar, Ronald P. van Rij

**Affiliations:** 1 Department of Medical Microbiology, Radboud University Medical Center, Radboud Institute for Molecular Life Sciences, P.O. Box 9101, 6500 HB Nijmegen, The Netherlands; 2 Center for Molecular and Biomolecular Informatics, Radboud University Medical Center, Radboud Institute for Molecular Life Sciences, P.O. Box 9101, 6500 HB Nijmegen, The Netherlands

## Abstract

Invertebrate RNA viruses are targets of the host RNA interference (RNAi) pathway, which limits virus infection by degrading viral RNA substrates. Several insect RNA viruses encode suppressor proteins to counteract this antiviral response. We recently demonstrated that the dsDNA virus Invertebrate iridescent virus 6 (IIV-6) induces an RNAi response in *Drosophila*. Here, we show that RNAi is suppressed in IIV-6-infected cells and we mapped RNAi suppressor activity to the viral protein 340R. Using biochemical assays, we reveal that 340R binds long dsRNA and prevents Dicer-2-mediated processing of long dsRNA into small interfering RNAs (siRNAs). We demonstrate that 340R additionally binds siRNAs and inhibits siRNA loading into the RNA-induced silencing complex. Finally, we show that 340R is able to rescue a Flock House virus replicon that lacks its viral suppressor of RNAi. Together, our findings indicate that, in analogy to RNA viruses, DNA viruses antagonize the antiviral RNAi response.

## INTRODUCTION

Recognition of double-stranded (ds) RNA is critical for many cellular processes, including gene regulation, RNA transport and RNA editing. Most dsRNA-protein interactions are established by proteins that contain a canonical dsRNA-binding domain (dsRBD), which binds dsRNA in a sequence-independent manner (1,2). RNA interference (RNAi) is a dsRNA-initiated mechanism for post-transcriptional gene silencing that is guided by small interfering RNAs (siRNAs) and requires dsRBD-containing proteins at several stages (3,4).

The RNAi pathway serves as a cellular defense mechanism that destroys viral RNA in diverse eukaryotes, including plants, fungi, nematodes, insects and mammals (5–8). In *Drosophila*, cytoplasmic Dicer-2 (Dcr-2), which contains two RNase III motifs and a single dsRBD, recognizes viral dsRNA as non-self and processes it into viral siRNAs (vsiRNAs), RNA duplexes of 21 nt that contain 2 nt 3′ overhangs (5,9). A heterodimer composed of the dsRBD adaptor protein R2D2 and Dcr-2 subsequently binds vsiRNA duplexes to mediate their loading into Argonaute-2 (AGO2) in the RNA-induced silencing complex (RISC). Within RISC, vsiRNAs guide the recognition and cleavage of fully complementary viral target RNA by AGO2 (9,10).

Several insect RNA viruses have evolved viral suppressors of RNAi (VSRs) to antagonize the initiation phase of the antiviral RNAi pathway (9). For example, the 1A protein of Drosophila C virus (DCV) contains a canonical dsRBD that binds long dsRNA and prevents Dcr-2-mediated vsiRNA biogenesis (11). The B2 protein of Flock House virus (FHV) and the VP3 proteins of Drosophila X virus and the mosquito-specific Culex Y virus interfere with the insect RNAi pathway by sequestering long dsRNA as well as siRNAs (12–16). These VSRs may thus inhibit the production of vsiRNAs and prevent their incorporation into RISC.

In RNA virus-infected plants, viral dsRNA is processed by the Dicer-like (DCL) proteins DCL2, DCL3 and DCL4, which generate viral small RNAs of 22-, 24- and 21-nt in size, respectively (5). The P19 protein of tombusviruses is one of the best-characterized VSRs in plants. P19 specifically binds 21-nt sized siRNAs (17–20) and thereby prevents siRNA incorporation into RISC (21). A similar strategy is used by several other plant RNA viruses (22,23). Sweet potato chlorotic stunt virus prevents siRNA loading into RISC via an alternative mechanism. This single-stranded RNA virus encodes a viral RNase III protein that processes siRNAs into 14-bp small RNA duplexes, which are non-functional in RNAi (24). Likewise, the RNase III of the dsDNA virus Heliothis virescens ascovirus-3e cleaves long dsRNA in the tobacco budworm (*Heliothis virescens*) (25). Thus, the RNase III proteins of two unrelated viruses interfere with RNAi, either by inhibiting vsiRNA production through destruction of Dcr-2 substrates or by preventing the incorporation of functional vsiRNAs into RISC.

Importantly, some insect and plant RNA viruses inhibit the RNAi pathway at the effector phase (9,23). For example, the Cricket paralysis virus (CrPV) 1A and Nora virus VP1 proteins antagonize the enzymatic activity of AGO2 in *Drosophila* (26,27). Similarly, the Cucumber mosaic virus 2b protein inhibits the endonuclease activity of AGO1 in plants (28). Plant AGO1 function is also suppressed by the P0 and P38 proteins of Beet western yellows virus and Turnip crinkle virus, respectively (29–32). These studies indicate that unrelated viruses have evolved VSRs that inhibit the catalytic activity of RISC and thus highlight the critical role of Argonaute proteins in antiviral defense.

Over the last years, several studies revealed that different classes of RNA viruses are both targets and suppressors of RNAi in *Drosophila* (11,13,33,34). Recently, we and others showed that RNAi also provides antiviral defense against DNA viruses *in vivo* (35,36). Indeed, Dcr-2-dependent vsiRNAs were generated in Invertebrate iridescent virus 6 (IIV-6)-infected flies and, accordingly, *AGO2* and *Dcr-2* mutant flies were more susceptible to IIV-6 infection than wild-type (WT) flies. However, it remained unknown whether DNA viruses antagonize the *Drosophila* antiviral RNAi response.

In the present study, we investigated whether IIV-6 suppresses RNAi. We demonstrate that the IIV-6 340R protein inhibits RNA silencing when RNAi is induced by long dsRNA as well as by siRNA duplexes. In a series of biochemical assays, we further demonstrate that 340R binds RNA duplexes to prevent siRNA biogenesis and to inhibit RISC loading. Our findings indicate that DNA viruses are targets and suppressors of the antiviral RNAi response.

## MATERIALS AND METHODS

### Cells and viruses

*Drosophila melanogaster* S2 cells were cultured as described previously (27). DCV and IIV-6 were propagated and titered as described previously (11,35).

### Plasmids

A proteinase K-treated IIV-6 virus stock was used as a template to amplify the 340R and 142R coding sequences, using primers that contain flanking XbaI restriction sites and introduce a *Drosophila* Kozak sequence (Supplementary Table S1). PCR products were subsequently cloned into the XbaI site of pAc5-V5-His B (Life Technologies), yielding plasmids that encode C-terminal V5 epitope-tagged proteins. Open reading frame (ORF) 340R mutant plasmids were generated by site-directed mutagenesis using the primers from Supplementary Table S1. The orientation and sequence of the selected clones was confirmed by DNA sequencing. Plasmids pAWH CrPV-1A, pMT-Luc and pMT-Ren were described previously (11,26). The pMT *Renilla* hairpin plasmid was kindly provided by R. Zhou (37). Plasmids encoding FHV replicons were described previously (16).

Plasmids encoding maltose-binding protein (MBP) fusion proteins were generated for the production of recombinant protein in *Escherichia coli*. The sequences coding for DCV 1A, WT 340R and the 340R mutants K89A and dsRBD^100^ were polymerase chain reaction (PCR) amplified and cloned as EcoRI-SalI fragments into pMAL-C2X (New England Biolabs) (see Supplementary Table S1 for primer sequences).

### RNAi reporter assays in S2 cells

The ability of IIV-6 to suppress RNAi-mediated silencing of firefly luciferase (Fluc) expression was analyzed as previously described for DCV (11). Briefly, 2.5 × 10^4^ S2 cells were seeded in a 96-well plate and mock-infected or infected with IIV-6 or DCV at an multiplicity of infection (MOI) of 0.1 or 0.01. Twenty-four hours after infection, cells were co-transfected with 12.5 ng pMT-Luc, 3 ng pMT-Ren, 50 ng empty pAc5-V5-His B plasmid and 10 ng dsRNA targeting either Fluc (dsFluc) or green fluorescent protein (GFP) (non-specific control, dsCtrl), using Effectene Transfection Reagent (Qiagen). Twenty-four hours after transfection, expression of the Fluc and *Renilla* luciferase (Rluc) reporters was induced by addition of 0.5 mM CuSO_4_ to the culture supernatant. Cell lysates were prepared after an additional 18-h incubation and luciferase activities were measured using the Dual luciferase reporter system (Promega).

Reporter assays in which RNAi was induced by dsRNA feeding were performed in S2R+ cells in a 96-well format. 3.0 × 10^4^ S2R+ cells were seeded and transfected the next day with 12.5 ng pMT-Luc, 3 ng pMT-Ren and either 50 ng pAc-VSR to express one of the viral proteins or the empty pAc vector. Two days after transfection, 400 ng dsRNA was added to the culture medium. Expression of reporter genes was induced at 8 h after dsRNA treatment and luciferase activities were measured the next day (38).

RNAi reporter assays in which RNAi was induced by *Renilla* hairpin RNA were performed in S2 cells. 3.0 × 10^5^ S2 cells were seeded in a 24-well plate and transfected the next day with 12 ng pMT-Ren, 50 ng pMT-Luc, 200 ng pAc-VSR plasmid and either with 75 ng of copper-inducible pMT hairpin-*Renilla* plasmid or, as non-silencing control, empty pMT plasmid. Expression of the *Renilla* hairpin RNA and the luciferase reporters was induced 2 days post-transfection by addition of copper sulfate to the culture supernatant and luciferase activities were measured at 18 h post-induction.

For the sequential co-transfection, 3.0 × 10^5^ S2 cells were seeded in 24-well plates. The next day, S2 cells were transfected with 100 ng pCoBlast (Life Technologies) and 300 ng of pAc-VSR plasmid. Forty-eight hours after transfection, the cells were transferred to 96-well plates in medium containing 25 μg/ml of blasticidin S (Life Technologies) to select for cells that express the viral proteins. The next day, a second transfection was performed with 12.5 ng pMT-Luc, 3 ng pMT-Ren, 50 ng pAc-empty carrier plasmid and 2 pmol of Fluc-specific siRNA (siFluc) or non-silencing control siRNA (siCtrl). The reporters were induced 24 h post-transfection and luciferase activities were measured the next day. For all reporter assays in which Fluc expression was silenced, Fluc counts were normalized to Rluc counts and expressed as fold silencing relative to control (empty vector) treatment, and vice versa when Rluc expression was silenced (38).

### Western blot analysis

To analyze protein expression from VSR expression plasmids, 3.0 × 10^5^ S2 cells were seeded in a 24-well plate. Twenty-four hours after seeding, cells were transfected with 500 ng of a VSR expression plasmid or an empty control plasmid using Effectene Transfection Reagent (Qiagen) and harvested at 3 days post-transfection. To analyze protein expression from VSR expression plasmids in our RNAi reporter assays, we pooled the cell lysates of 10 individual wells of a 96-well plate. Proteins were separated on a 12.5% denaturing polyacrylamide gel and transferred to an 0.2-μm nitrocellulose membrane (Bio-Rad). The membrane was stained by subsequent incubations in anti-V5 mouse monoclonal antibodies (Life Technologies) and Alexa Fluor 680-conjugated goat anti-mouse antibodies (LI-COR Biosciences). As a loading control, the same membrane was probed with anti-α-tubulin antibodies (AbD Serotec) and Alexa Fluor 800-conjugated goat anti-rat antibodies (LI-COR Biosciences). Bound antibodies were visualized on an Odyssey infrared imager (LI-COR Biosciences).

### Homology modeling

To predict the protein structure of 340R, we generated a homology model using the YASARA & WHAT IF Twinset under default settings (39,40). The experimentally solved protein structure of TRBP2 (Protein database accession 3ADL) and *Aquifex aeolicus* RNase III (PDB 2NUG) were used as a template (41,42). The model contained residues 1–112 of 340R (out of a total length of 173 aa), of which residues 1–37 were modeled after Aa-RNase III and residues 20–112 after TRBP2.

### Production and purification of recombinant proteins

Plasmids encoding MBP fusion proteins were transformed into the *E. coli* BL21 (DE3) strain and expression of recombinant proteins was induced with 1 mM isopropyl β-D-1-thiogalactopyranoside (IPTG) at an OD_600_ of 1.2. The cultures were incubated for 3 h at 37°C for pMAL-empty and pMAL-DCV 1A and for 4.5 h at 25°C for pMAL-340R. Fusion proteins were affinity-purified using amylose resin according to the manufacturer's protocol (New England Biolabs). Recombinant proteins were transferred to a dialysis membrane (molecular weight cut-off 12–14 kDa) and dialyzed to buffer (20 mM Tris-HCl, 0.5 mM ethylenediaminetetraacetic acid, 5 mM MgCl_2_, 1 mM DTT, 140 mM NaCl, 2.7 mM KCl). Recombinant proteins were stored as aliquots at –80°C in dialysis buffer containing 30% glycerol. Protein concentrations were determined by a Bradford assay (Bio-Rad).

### Electrophoretic mobility shift assays (EMSAs)

Radiolabeled probes for EMSAs were generated as described before (43). Uniformly labeled 126-nt blunt dsRNA was generated by *in vitro* transcription of T7 promoter-flanked PCR fragments using T7 RNA polymerase in the presence of α-^32^P-[UTP]. After annealing of the two radiolabeled RNA strands, unincorporated nucleotides were removed using a G-25 Sephadex column (Roche) and dsRNA was purified from an 8% native polyacrylamide gel. Synthetic 21-nt siRNAs containing 2-nt 3′ overhangs and 19-nt blunt dsRNAs (43) were end-labeled with γ-^32^P-[ATP] using T4 polynucleotide kinase (Roche) and purified on a G-25 Sephadex column.

EMSAs were performed as described previously (11). Briefly, radiolabeled 126-nt long dsRNA (5 ng), 19-nt dsRNA or siRNA duplexes (1 nM) were incubated with different concentrations of recombinant proteins for 1 h at room temperature. Long dsRNA and siRNA EMSAs were analyzed on 6% and 12% native polyacrylamide gels, respectively. Gels were exposed to a Kodak Biomax XAR film and radioactive signals were quantified with ImageJ software.

### Dicer and slicer assays

To analyze processing of long dsRNA into siRNA, we performed *in vitro* Dicer assays in *Drosophila* S2 cell lysate as described before (27,44). 11 × 10^6^ S2 cells were seeded in T75 flasks and 1 day after seeding, cells were either mock-infected or infected with IIV-6 at an MOI of 1.0 or 0.1. Two days post-infection, cells were harvested and homogenized in lysis buffer [30 mM HEPES-KOH, 100 mM KOAc, 2 mM Mg(OAc)_2_, 5 mM DTT and complete protease inhibitor mixture (Roche)] for 1 h on ice. Protein concentrations of S2 cell extracts were analyzed by a Bradford assay (Bio-Rad) and lysates were frozen at –80°C. Before analyzing Dicer activity, cell extracts were thawed on ice and centrifuged for 30 min at 16 000 × *g* at 4°C to remove cell debris.

To analyze AGO2 target RNA cleavage, we performed *in vitro* Slicer assays in *Drosophila* embryo lysates as described previously (27,44).

### FHV replicon assay

S2 cells were seeded in a 24-well plate at a density of 3.0 × 10^5^ cells per well. The next day, cells were transfected with 100 ng of plasmid encoding either the WT FHV replicon or the B2-deficient replicon (FHV ΔB2) along with either 300 ng of pAc-VSR plasmid or empty control plasmid. Two days after transfection, 0.5 mM CuSO_4_ was added to the culture medium to induce transcription of the FHV replicon. The following day, cells were harvested and total RNA was isolated using Isol-RNA Lysis reagent. RNA was treated with DNase I (Life Technologies) and cDNA synthesis was performed using TaqMan Reverse Transcription Reagents (Life Technologies) and a strand-specific FHV primer tagged with a 5′ T7 promoter sequence (43). Following cDNA synthesis, qPCR analysis was performed using a combination of a T7 promoter primer and an FHV-specific forward primer. Data were normalized to Rp49 (RpL32), for which strand-specific quantitative reverse transcriptase-PCR (qRT-PCR) assays were run in parallel (43), and presented as fold change relative to empty vector control.

## RESULTS

### RNAi is suppressed in IIV-6-infected cells

We and others recently reported that the dsDNA virus IIV-6 triggers an antiviral RNAi response in *Drosophila* (35,36). To investigate whether IIV-6 antagonizes the host RNAi response, we performed RNAi reporter assays in *Drosophila* S2 cells. In these well-established assays, RNAi-mediated silencing of a Fluc reporter gene is induced by Fluc-specific long dsRNA or siRNAs (11,38).

**Figure 1.**
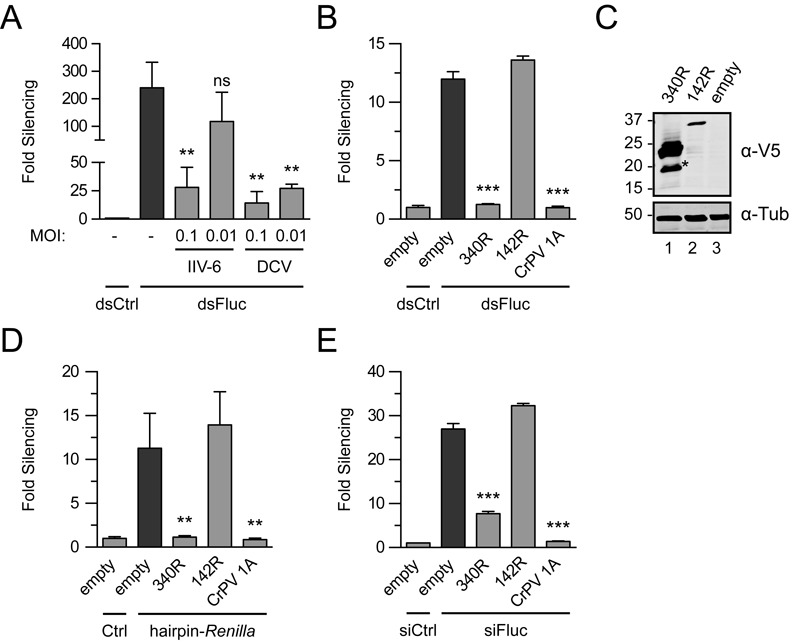
IIV-6 encodes a suppressor of RNAi. (A) dsRNA-induced RNAi reporter assay in virus-infected *Drosophila* S2 cells. S2 cells were mock-infected (-) or infected with either IIV-6 or DCV at the indicated MOI. Fluc and *Renilla* luciferase (Rluc) reporter plasmids were transfected along with dsRNA targeting Fluc (dsFluc) or a non-specific control sequence (dsCtrl) and 3 days after transfection, luciferase activities were measured. (B) RNAi reporter assay in S2R^+^ cells expressing individual viral proteins. Cells were transfected with luciferase reporter plasmids along with control plasmid (empty) or expression plasmids encoding the indicated viral proteins. CrPV 1A was included as a positive control. Two days post-transfection, RNAi was induced by adding long dsRNA to the culture supernatant. (C) Western blot analysis of V5 epitope-tagged 340R and 142R in transfected S2 cells. Viral proteins were detected using anti-V5 antibodies (α-V5); polyclonal anti-α-tubulin (α-Tub) antibody was used as a loading control. Molecular mass (in kDa) is indicated on the left of the image. The predicted molecular weights for 340R and 142R are 23 and 37 kDa, respectively. The asterisk (*) indicates a 340R-specific processing or degradation product. (D) *Renilla* hairpin-induced RNAi reporter assay. S2 cells were transfected with luciferase reporter plasmids, expression plasmids for the indicated viral proteins and either a plasmid that encodes a *Renilla* hairpin RNA (hairpin-*Renilla*) or an empty plasmid control (Ctrl). (E) siRNA-induced RNAi reporter assay in S2 cells. Cells were transfected with plasmids encoding the indicated viral proteins followed by transfection of luciferase reporter plasmids along with siRNAs that target the Fluc reporter (siFluc) or non-silencing control siRNAs (siCtrl). For all reporter assays in which Fluc expression was silenced, Fluc counts were normalized to Rluc counts and expressed as fold silencing relative to control treatment and vice versa when Rluc expression was silenced. Fold silencing in the non-silencing controls (dsCtrl or siCtrl) was set to one for all panels. Bars in all panels represent the means and standard deviations of three independent samples. Difference in RNAi efficiency compared to controls (dark gray bars) was analyzed by one-way ANOVA followed by a *post hoc* Dunnett's test. *P ≤ 0.05; **P ≤ 0.01; ***P ≤ 0.001; ns, not significant.

We first tested whether RNAi is suppressed in IIV-6-infected S2 cells. As a positive control, we included the positive-sense RNA virus DCV, which encodes a VSR and inhibits RNAi in infection (11). Co-transfection of reporter plasmids along with Fluc-specific long dsRNA resulted in efficient silencing of the Fluc reporter in mock-infected cells (240-fold, Figure 1A). In contrast, in IIV-6-infected cells, silencing of the Fluc reporter was suppressed in an MOI-dependent manner, to 118-fold at an MOI of 0.01 and to 28-fold at an MOI of 0.1 (Figure 1A; *P* = 0.061 and *P* = 0.004, respectively). As observed before (11), DCV also suppressed RNAi in an MOI-dependent manner (Figure 1A). Together, these results indicate that the RNAi pathway is suppressed in IIV-6-infected cells.

### The IIV-6 340R protein is a suppressor of RNAi

IIV-6 is a large, complex virus with a 212-kb genome that contains 211 predicted ORFs (45,46). We therefore browsed the IIV-6 genome for ORFs that encode proteins with predicted domains or motifs that might account for the observed RNAi suppressor activity. A candidate is ORF 142R, which encodes a putative RNase III that might degrade siRNAs or long dsRNA substrates for Dcr-2, as was observed for RNase III proteins of other viruses (24,25). Another candidate VSR is 340R, which contains a predicted canonical dsRBD. Such domains have also been observed in other VSRs (11,47). The 142R and 340R proteins are conserved in different genera within the *Iridoviridae* family (46,48), suggesting that these proteins have important functions in the viral life cycle.

To analyze whether 142R and 340R inhibit the RNAi pathway, we cloned the individual ORFs into expression plasmids for RNAi reporter assays. S2 cells were transfected with the expression plasmids along with the reporter plasmids. Two days after transfection, dsRNA was added to the culture supernatant to induce RNAi, thus allowing expression of viral proteins before induction of RNAi. The CrPV 1A protein, which suppresses the RNAi pathway by antagonizing RISC enzymatic activity, served as a positive control (26). The dsRBD protein 340R suppressed RNAi to background levels (compared with 12-fold silencing for the empty control vector, *P* ≤ 0.001), similar to CrPV 1A (Figure 1B, *P* ≤ 0.001). In contrast, we did not observe VSR activity for the predicted RNase III 142R (Figure 1B). Although we readily detected protein expression of 142R and 340R in transfected S2 cells by western blot analysis (Figure 1C), 142R was expressed at lower levels than 340R. Increasing the amount of transfected 142R expression plasmid led to a mild increase in protein levels. However, also under these conditions, we could not detect VSR activity for 142R (Supplementary Figure S1A). To confirm these results, we performed an RNAi reporter assay in which a *Renilla* luciferase (Rluc) reporter is silenced by an Rluc-specific hairpin RNA expressed from a copper-inducible promoter. Also in this assay, 340R efficiently suppressed RNAi (Figure 1D, *P* = 0.003), whereas 142R was unable to do so.

To investigate whether 340R inhibits the RNAi pathway downstream of siRNA production, we performed an assay in which we induced RNAi with siRNAs instead of long dsRNA. We found that 340R efficiently inhibited siRNA-induced RNAi (Figure 1E; 7.7-fold silencing compared with 27-fold for the empty control plasmid, *P* ≤ 0.001), indicating that this VSR is capable of suppressing the RNAi pathway at a stage downstream of Dcr-2-dependent siRNA production.

### The dsRBD of 340R is required for RNAi suppression

**Figure 2.**
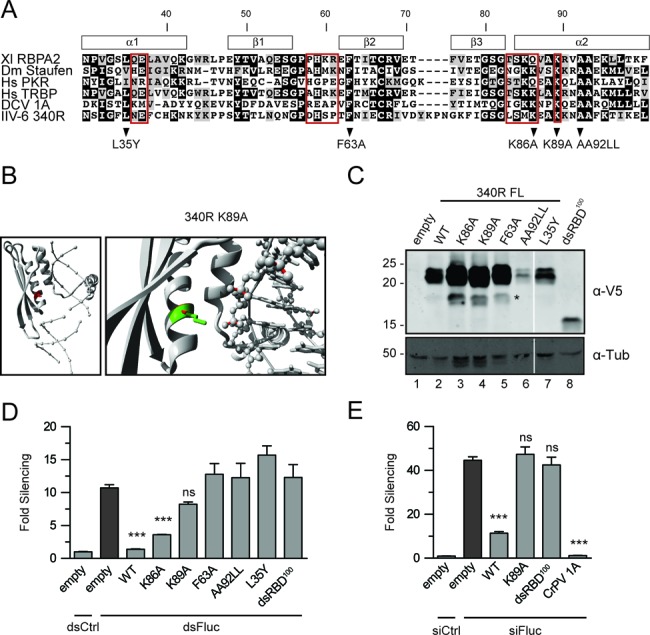
Viral protein 340R requires a functional dsRBD to suppress RNAi. (A) Alignment of the dsRBD of 340R (aa 30–100) to the dsRBDs of proteins from different model organisms and to the dsRBD of DCV 1A. Red boxes indicate residues that interact with dsRNA in structural analyses of the second dsRBD of *Xenopus laevis* RNA-binding protein A (Xl RBPA2) (56). Residues predicted to be involved in RNA binding or conserved amino acids were selected for site-directed mutagenesis. Position of secondary structures are indicated above the alignment. (B) Homology model of 340R in complex with dsRNA. The protein is shown in cartoon-view with the WT residue K89 shown in red (left panel) or with the WT residue shown in green and the mutant residue (Alanine) shown in red (right panel). The RNA is shown in ball-and-stick view, without atomic details (left) and with all atoms (right). The side-chain of residue K89 is positioned toward the phosphate backbone of the RNA and likely binds dsRNA through an electrostatic interaction of the positively charged Lysine with the negatively charged phosphates. The substitution of this Lysine into the small and hydrophobic Alanine is likely to abolish the interaction. See Supplementary Figure S2 for other residues selected for site-directed mutagenesis. (C) Western blot analysis of V5 epitope-tagged WT and mutant 340R from transfected S2 cells. Proteins were detected using anti-V5 antibodies (α-V5) or, as a loading control, using anti-α-tubulin (α-Tub) antibodies. Molecular mass (in kDa) is indicated on the left of the image. The predicted molecular weight for dsRBD^100^ is 14.7 kDa. FL, full-length. The asterisk (*) indicates a 340R-specific processing or degradation product. (D) dsRNA-induced RNAi reporter assay. The experiment was performed as described in the legend to Figure 1B, using expression plasmids for WT and mutant 340R. (E) siRNA-induced RNAi reporter assay. Sequential transfection was performed as described in the legend to Figure 1E, using expression plasmids for WT and mutant 340R and CrPV 1A. Difference in RNAi efficiency compared to controls (dark gray bars) was analyzed by one-way ANOVA followed by a *post hoc* Dunnett's test. *P ≤ 0.05; **P ≤ 0.01; ***P ≤ 0.001; ns, not significant.

The IIV-6 340R gene encodes a 23-kDa protein that contains a 70-aa dsRBD flanked by a 30-aa N-terminal sequence and a 73-aa C-terminal sequence. Alignment of the the dsRBD of 340R to the dsRBDs of DCV 1A and cellular proteins from different model organisms shows that conserved amino acids are present throughout the motif (Figure 2A). Homology modeling suggests that the dsRBD of 340R adopts the expected αβββα topology, in which two α helices are packed along a three-stranded antiparallel β sheet, and that the dsRBD is preceded by an N-terminal helical structure (Figure 2B). Based on these analyses, we selected for site-directed mutagenesis four highly conserved residues (L35Y, F63A and AA92LL) and two residues within a region expected to interact with dsRNA (K86A and K89A) (Figure 2A and B and Supplementary Figure S2). In addition, we generated a C-terminally truncated version of 340R, consisting of the N-terminal 100-aa that contains the complete dsRBD (dsRBD^100^).

Western blot analysis verified that all mutant proteins were expressed in transfected S2 cells, albeit at different levels relative to the WT protein (Figure 2C). We subsequently analyzed VSR activity of WT and mutant 340R in reporter assays in which RNAi was induced by feeding of long dsRNA. In these assays, WT 340R almost completely blocked RNAi (Figure 2D; 1.4-fold silencing compared with 11-fold for the empty control vector, *P* ≤ 0.001). All tested mutants lost VSR activity relative to WT 340R (Figure 2D). Mutation of residues predicted to be involved in dsRNA binding reduced silencing to 3.6-fold (K86A, *P* ≤ 0.001) and 8.2-fold (K89A, *P* = 0.074) (Figure 2D). Loss of VSR activity of the conserved residue mutants AA92LL and L35Y might result from reduced expression levels (Figure 2C), perhaps due to destabilizing effects of the substitutions on the local protein structure (Supplementary Figure S2).

To increase VSR protein levels in our RNAi reporter assays, we increased the amount of transfected L35Y and AA92LL expression plasmids (Supplementary Figure S2B and S2C, respectively). L35Y expression was increased to WT 340R levels under several conditions, but this did not result in detectable VSR activity (Supplementary Figure S2B). For the AA92LL mutant, we only observed a slight increase in protein levels, which was not sufficient for suppression of RNAi (Supplementary Figure S2C). Similar to the 340R point mutants, dsRBD^100^ did not suppress RNAi (Figure 2D). Since this construct was expressed at lower levels than WT 340R, we repeated the assay with increasing amounts of plasmid. However, although dsRBD^100^ reached similar protein levels as WT 340R under these conditions, dsRBD^100^ did not inhibit silencing of the reporter, indicating that the dsRBD by itself is insufficient to exert VSR activity (Supplementary Figure S1D).

We next performed an RNAi reporter assay in which RNAi is induced by siRNA transfection and included dsRBD^100^ as well as the K89A mutant (Figure 2E). As observed before (Figure 1E), WT 340R, as well as the positive control CrPV 1A, efficiently suppressed siRNA-induced RNAi (11- and 1.2-fold silencing, respectively, compared with 44-fold for the empty vector). The K89A and dsRBD^100^ mutants completely lost VSR activity (47- and 43-fold silencing, respectively), suggesting that suppression of siRNA-induced RNAi requires the dsRNA-binding activity of 340R as well as its 73-aa C-terminal domain.

### 340R binds long dsRNA and siRNA duplexes

**Figure 3.**
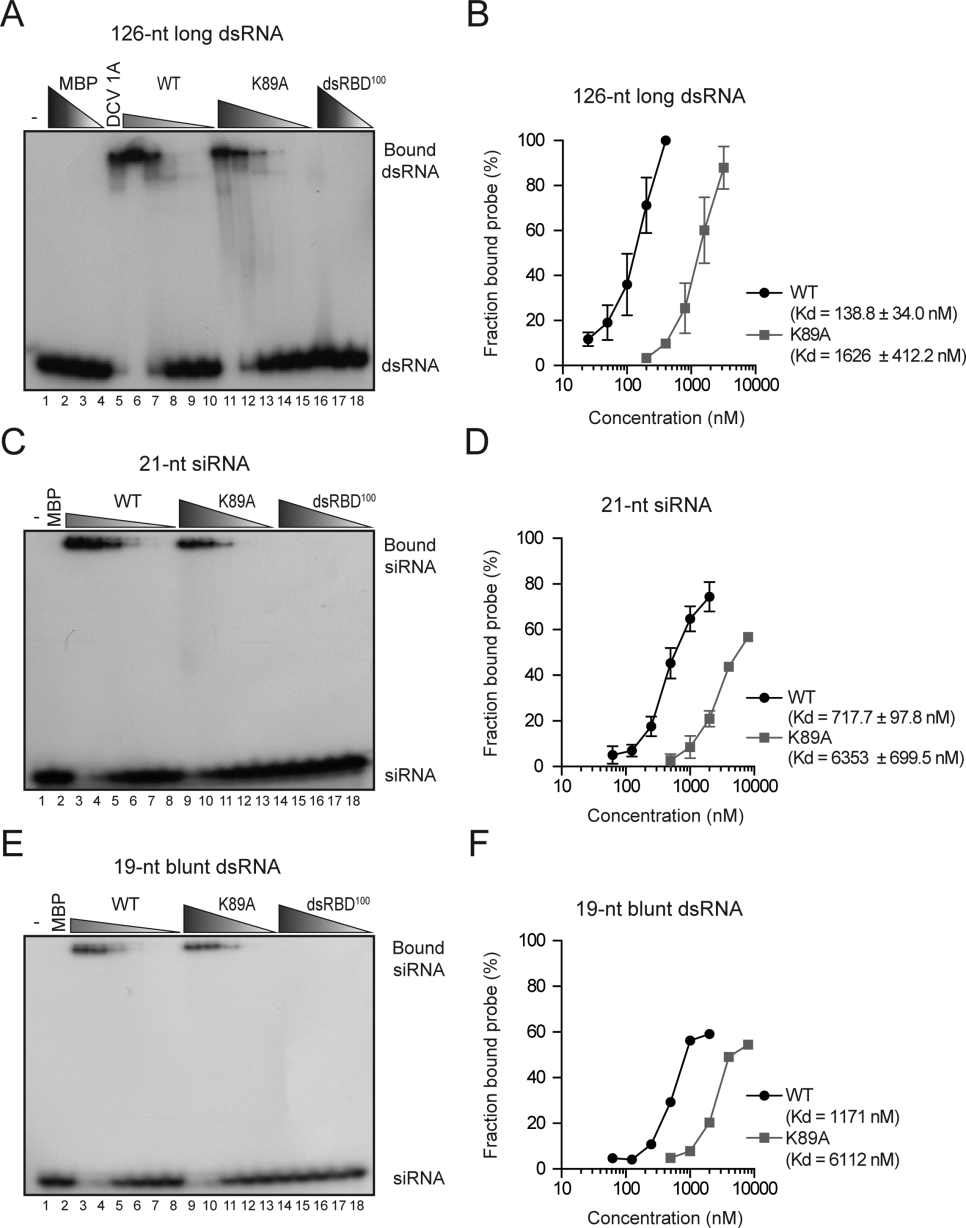
340R binds long dsRNA and duplex siRNAs. (A) EMSA of 126-nt blunt dsRNA with WT 340R and the K89A and dsRBD^100^ mutants. Buffer only (-, lane 1) and decreasing concentrations of MBP were included as negative controls (lanes 2–4; 10, 3.2 and 0.4 μM). An MBP-DCV 1A fusion protein was included as positive control (lane 5; 0.1 μM). WT and K89A 340R were tested in 2-fold serial dilutions starting at 0.4 μM (WT, lanes 6–10) and 3.2 μM (K89A, lanes 11–15). dsRBD^100^ 340R protein was tested in 10-fold dilutions starting at 10 μM (lanes 16–18). (B) Quantification of the fraction bound probe at different protein concentrations for WT 340R (black line) and the K89A mutant (gray line). Data represent means and standard deviations of three independent experiments. (C) EMSA of 21-nt siRNAs containing 2-nt 3′ overhangs with MBP (lane 2; 8 μM) and 2-fold serial dilutions of WT 340R (lanes 3–8; starting at 2 μM), and the K89A and dsRBD^100^ mutants (lanes 9–13 and 14–18, respectively; starting at 8 μM). A representative experiment of three independent experiments is shown in panels A and C. (D) Quantification of the fraction bound siRNA at different protein concentrations for WT 340R (black line) and the K89A mutant (gray line). Data represent means and standard deviations of three independent experiments. (E) EMSA of 19-nt blunt dsRNA with decreasing amounts of recombinant proteins. Protein concentrations are as described in panel (C). (F) Quantification of the fraction bound 19-nt blunt dsRNA probe at different protein concentrations for WT 340R (black line) and the K89A mutant (gray line).

To directly analyze dsRNA binding by 340R, we performed EMSAs using different dsRNA substrates. Full-length WT 340R and the VSR-defective mutants K89A and dsRBD^100^ were fused to MBP and affinity-purified from *E. coli*. We first tested whether these recombinant proteins can bind radioactively labeled, 126-bp long dsRNA. As positive control, we included recombinant MBP-DCV 1A. As expected, no shift in mobility of long dsRNA on native polyacrylamide gels was observed with increasing concentrations of MBP (Figure 3A, compare lanes 2–4 with lane 1). By contrast, addition of DCV 1A resulted in protein-dsRNA complex formation (Figure 3A, lane 5), which is in line with previous observations (11,27). Similarly, WT 340R bound dsRNA in a dose-dependent manner (Figure 3A, lanes 6–10). Interestingly, the K89A mutant could still bind long dsRNA, although 8-fold higher protein concentrations were required for a complete dsRNA shift (Figure 3A, lanes 11–15). Indeed, WT 340R had a ∼12-fold higher affinity for long dsRNA than the K89A mutant (dissociation constants of 138.8 ± 34.0 nM and 1626 ± 412.2 nM, respectively, Figure 3B). No dsRNA-binding activity was detected for dsRBD^100^, even when a 25-fold higher protein concentration was tested (Figure 3A, lanes 16–18). These results are in line with the results from the RNAi reporter assay, in which we observed slight VSR activity for the K89A mutant and a lack of VSR activity for dsRBD^100^ (Figure 2D).

We next used EMSAs to analyze whether 340R has binding affinity for siRNA duplexes. Synthetic 21-nt siRNA duplexes that contain 2-nt 3′ overhangs shifted after incubation with increasing amounts of WT 340R (Figure 3C, lanes 3–8) and the K89A mutant (Figure 3C, lanes 9–13), indicating that these proteins are able to bind siRNAs. No protein-siRNA complexes were formed with either MBP (Figure 3C, lane 2) or dsRBD^100^ (Figure 3C, lanes 14–18). Similar to the long dsRNA binding assay, higher concentrations of the K89A mutant were required to observe an siRNA shift. Accordingly, WT 340R had higher affinity for siRNA duplexes than the K89A mutant (dissociation constants of 717.7 ± 97.8 nM and 6353 ± 699.5 nM, respectively, Figure 3D).

To analyze whether the 3′ overhangs are required for efficient siRNA binding, we used a 19-nt blunt dsRNA probe in EMSAs (Figure 3E). These experiments revealed that both WT and K89A mutant 340R bind 19-nt blunt dsRNA in a dose-dependent manner (Figure 3E, lanes 3–8 and 9–13, respectively), with dissociation constants of 1171 and 6112 nM, respectively (Figure 3F). The observation that both WT and K89A mutant 340R had similar binding affinities for siRNAs and 19-nt blunt dsRNA (Supplementary Figure S3), indicate that the 2-nt 3′ overhangs of siRNAs are not essential for efficient siRNA binding. Taken together, these results show that WT 340R efficiently binds both long and short dsRNA, as well as siRNA duplexes.

### 340R inhibits Dcr-2-dependent dsRNA processing

**Figure 4.**
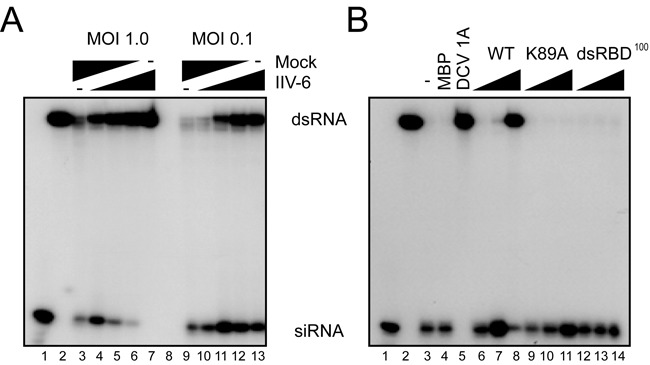
340R inhibits Dicer-dependent production of siRNAs. (A) Processing of radiolabeled long dsRNA into siRNAs in cytoplasmic extracts of S2 cells that were mock-infected or infected with IIV-6 at the indicated MOI. siRNA production was analyzed in lysates of mock-infected cells (lanes 3 and 9), IIV-6-infected cells (lanes 7 and 13) and in mixtures of infected and non-infected lysates at different ratios (1:3; 1:1; 3:1; lanes 4–6 and 10–12). Synthetic siRNA and unprocessed dsRNA were loaded on gel as size markers (lanes 1 and 2, respectively). (B) Processing of dsRNA into siRNAs was analyzed in non-supplemented S2 cell extract (lane 3), and in cell extracts supplemented with recombinant MBP (lane 4; 1 μM), DCV 1A (lane 5; 1 μM) and increasing concentrations of WT 340R (lanes 6–8), and the K89A (lanes 9–11) and dsRBD^100^ (lanes 12–14) mutants (0.01, 0.1 and 1 μM). Size markers for siRNA and dsRNA were loaded in lanes 1 and 2, respectively.

Our data show that 340R interacts with dsRNA and that the dsRBD and C-terminus are required for its VSR activity. The dsRNA-binding activity of 340R may inhibit RNAi at two stages. First, by binding to dsRNA, it may shield long dsRNA from processing by Dcr-2. Second, by sequestering siRNAs, it may prevent incorporation of small RNAs into RISC. We performed *in vitro* Dicer assays to test whether dsRNA processing is inhibited in lysates of IIV-6-infected cells. In these assays, we analyzed cleavage of a radiolabeled 126-nt dsRNA substrate into 21-nt siRNAs on denaturing polyacrylamide gels. In mock-infected cell lysates, dsRNA was efficiently processed into siRNAs (Figure 4A, lane 3), whereas siRNA production was completely blocked in lysates from IIV-6-infected cells (MOI of 1.0) (Figure 4A, lane 7). Using a mixture of IIV-6 and mock-infected cell lysates at different ratios, dsRNA processing was inhibited in a dose-dependent manner (Figure 4A, lanes 4–6). Similar results were observed in lysates from cells that were infected with IIV-6 at an MOI of 0.1 (Figure 4A, lanes 9–13), albeit that Dicer activity was not completely blocked at this lower MOI (Figure 4A, compare lane 13 with lane 7). Together, these results indicate that IIV-6 encodes an inhibitor of dsRNA processing.

We next tested our hypothesis that the 340R protein interferes with Dcr-2-mediated dsRNA processing. Long dsRNA was efficiently processed into siRNAs in non-supplemented S2 cell extracts and in extracts supplemented with recombinant MBP (Figure 4B, lanes 3 and 4). WT 340R inhibited processing of long dsRNA in a dose-dependent manner (Figure 4B, lanes 6–8). Likewise, the addition of recombinant DCV 1A, a VSR that is known to bind long dsRNA (11), completely blocked dsRNA cleavage (Figure 4B, lane 5). In contrast, the K89A or dsRBD^100^ mutants could not prevent the production of siRNAs, even at the highest concentration tested (Figure 4B, lanes 9–11 and 12–14, respectively). It is worthwhile noting that the K89A mutant does show dsRNA-binding activity in EMSAs (Figure 3A, lanes 11–14), but that it does not protect dsRNA from Dicer-mediated processing (Figure 4B, lanes 9–11). Altogether, these data indicate that WT 340R interferes with Dcr-2-dependent siRNA biogenesis and that efficient dsRNA binding is required to prevent Dcr-2 processing activity.

### 340R does not inhibit AGO2 slicing activity

**Figure 5.**
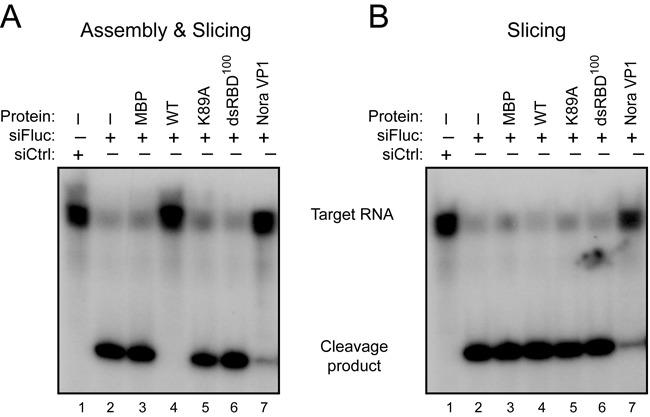
340R does not inhibit Slicer activity of pre-assembled RISC. (A) *In vitro* RNA cleavage (Slicer) assay in *Drosophila* embryo lysates to analyze the effect of 340R on RISC assembly and subsequent AGO2 catalytic activity. Embryo lysates were pre-incubated for 30 min with recombinant proteins (lanes 3–8) or protein storage buffer (lanes 1 and 2), followed by the addition of Fluc-specific siRNAs (siFluc, lanes 2–8) or non-specific control siRNAs (siCtrl, lane 1). After another 30-min incubation, a radioactive cap-labeled Fluc target RNA was added to the reaction mixture. Target cleavage was analyzed on a denaturing gel after a further 2-h incubation. (B) Slicer assay to monitor the effect of WT 340R on Slicer activity of a pre-assembled RISC. Recombinant proteins (lanes 3–8) were added after RISC assembly for 30 min with siFluc (lanes 2–4) or siCtrl (lane 1). After a further 30-min incubation, target RNA was added and the reaction was allowed to proceed for 2 h before analysis. Nora virus VP1 was analyzed at a concentration of 0.3 μM, all other proteins at 1.5 μM.

Having shown that 340R interferes with the initiation steps of the RNAi pathway, we wondered whether 340R also inhibits the effector phase of the RNAi response. We thus monitored slicing of a radioactively 5′ cap-labeled target RNA (44) in the presence or absence of 340R. *Drosophila* embryo lysates were incubated with a 492-nt Fluc target RNA sequence and a Fluc-specific siRNA that triggers target RNA cleavage into a 164-nt 5′ cleavage product (Figure 5A, lane 2). This specific cleavage product was not detected after incubation with a non-specific control siRNA (Figure 5A, lane 1). In a first approach, we analyzed whether 340R interferes with RISC assembly and subsequent target RNA cleavage. To this end, we incubated recombinant proteins with embryo lysate before the addition of siRNAs (27). Using this approach, we observed that WT 340R efficiently inhibited target RNA cleavage (Figure 5A, lane 4). In contrast, the K89A and dsRBD^100^ mutants did not suppress slicing (Figure 5A, lanes 5 and 6), similar to MBP alone (lane 3). To differentiate between the effect of 340R on RISC assembly and target slicing, we next tested whether WT 340R affects slicing by interfering with a pre-assembled RISC. To allow mature RISC formation, we pre-incubated embryo extracts with siRNAs before the addition of 340R. Neither MBP alone (Figure 5B, lane 3) nor WT or mutant 340R (lanes 4–6) inhibited target RNA cleavage under these conditions. In contrast, the positive control Nora virus VP1 efficiently inhibited AGO2-mediated target cleavage in both experimental approaches (Figure 5A and B, lane 7) (27). These results demonstrate that 340R does not inhibit the activity of a pre-assembled mature RISC. Because 340R binds siRNAs (Figure 3C and D), we propose that 340R interferes with the RISC assembly process by preventing siRNA loading into AGO2.

### IIV-6 340R rescues replication of a VSR-defective FHV replicon

**Figure 6.**
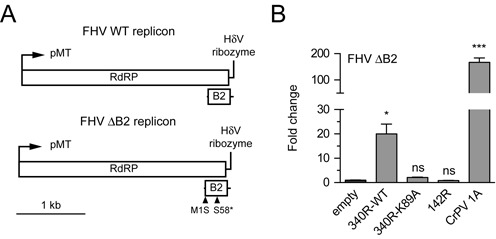
340R rescues VSR-deficient FHV replication. (A) Schematic representation of the WT and ΔB2 FHV replicons. FHV RNA1 is self-replicating and encodes the RNA-dependent RNA polymerase (RdRP). B2, the viral suppressor of RNAi, is expressed from a subgenomic RNA (upper panel). The FHV ΔB2 replicon lacks B2 expression due to point mutations (triangles) that disrupt the start codon (M1S) and introduce a premature stop codon (S58*) (bottom panel) (49). pMT, metallothionein promoter; HdV, Hepatitis delta virus. (B) FHV RNA levels in S2 cells co-transfected with the FHV ΔB2 replicon and expression plasmids encoding the indicated viral proteins. FHV RNA levels were analyzed by qRT-PCR, normalized to Rp49 and presented as fold change relative to the empty vector control. Bars represent the means and standard deviations of three independent samples. Viral RNA levels were compared to the control (empty plasmid) with one-way ANOVA followed by a *post hoc* Dunnett's test. *P ≤ 0.05; **P ≤ 0.01; ***P ≤ 0.001; ns, not significant.

To analyze whether the 340R-mediated VSR activity is sufficient to suppress an antiviral RNAi response, we investigated whether 340R can rescue replication of a VSR-defective FHV replicon (12,34). The WT replicon consists of RNA1 of FHV, which encodes the viral RNA-dependent RNA polymerase (RdRP) and expresses the B2 suppressor protein that antagonizes RNAi by binding dsRNA and siRNA duplexes (Figure 6A) (12,14,15). In the VSR-defective FHV replicon (FHV ΔB2), two-point mutations were introduced that abolish B2 expression, resulting in an RNAi-dependent replication defect (Figure 6A) (12,43,49).

We first analyzed whether CrPV 1A can rescue the replication defect of the FHV ΔB2 replicon. We performed RT-qPCR analysis to determine FHV RNA levels and observed that the FHV ΔB2 replicon replicates efficiently when CrPV 1A is expressed (∼170-fold increase over the empty vector control, Figure 6B), confirming that the FHV ΔB2 replicon is restricted by an AGO2-dependent antiviral RNAi response. Next, we tested whether WT 340R and the K89A mutant can rescue FHV ΔB2 replication. Upon expression of WT 340R, FHV RNA levels were 20-fold higher than in cells transfected with the empty vector control (Figure 6B, *P* = 0.018), whereas the K89A mutant was unable to rescue FHV ΔB2 replication (∼2-fold increase). In line with the results of the RNAi reporter assays (Figure 1), the putative RNase III 142R did not rescue FHV ΔB2 replication. Together, our results show that 340R suppresses the antiviral RNAi pathway.

## DISCUSSION

In recent years, it has become clear that different classes of RNA viruses are targets of the RNAi pathway in *Drosophila*. The antiviral activity of the RNAi machinery is mediated by Dcr-2-dependent cleavage of viral dsRNA into vsiRNAs that guide AGO2-dependent slicing of viral single-stranded RNA (9,11,13,26,33,34,50). During the ongoing arms race between viruses and their hosts, viruses have evolved sophisticated mechanisms to suppress or evade host-based immune responses. The best-studied examples of viral antagonism of RNAi in *Drosophila* come from studies on RNA viruses, which encode VSRs that interfere with the initiation and effector phases of the RNAi pathway (9). DNA viruses also induce an RNAi response in *Drosophila*, which is initiated by processing of viral dsRNA substrates derived from overlapping convergent transcripts (35,36) or from structured regions within viral transcripts (51,52). However, it remained unclear whether DNA viruses inhibit this small RNA-based immune response in *Drosophila*. In this study, we show that the dsRBD-containing protein 340R from the DNA virus IIV-6 suppresses RNAi.

IIV-6 is a nucleocytoplasmic virus that can infect *Drosophila*-derived cells as well as adult flies (35,36,53,54). This linear dsDNA virus contains 211 putative ORFs, which are transcribed from either strand of the viral genome (45,46). We show that RNAi is inhibited in IIV-6-infected cells and demonstrate that the IIV-6-encoded 340R protein inhibits Dcr-2 processing and RISC loading through duplex RNA binding. However, we cannot exclude the possibility that IIV-6 produces additional VSRs that contribute to RNAi antagonism. The plant RNA virus Citrus tristeza virus, for example, encodes three distinct VSRs to inhibit the antiviral RNAi response at different levels (55). Studies on *Xenopus laevis* RNA-binding protein (Xlrbp) and *Drosophila* Staufen revealed that their dsRBDs alone are sufficient to bind dsRNA (56–58). Surprisingly, however, the C-terminal deletion mutant dsRBD^100^, which contains the entire dsRBD, was unable to bind RNA duplexes and did not exert VSR activity. How the C-terminal region of 340R contributes to VSR activity remains an open question for further studies.

Viral RNase III proteins are conserved among all genera within the *Iridoviridae* family, suggesting that this protein has important functions within the viral life cycle (46). However, under our experimental conditions, the putative RNase III 142R did not show VSR activity in reporter assays and was not able to rescue FHV ΔB2 replication. These observations suggest that the IIV-6-encoded RNase III is not involved in suppression of the RNAi response. This is in contrast to the proposed VSR activity of the RNase III proteins from Heliothis virescens ascovirus-3e, a DNA virus that infects moths, and from the plant RNA virus Sweet potato chlorotic stunt virus (24,25). 142R is structurally similar to bacterial RNase III proteins that are involved in the processing of structured, non-coding RNAs and specific mRNAs (59). Similarly, Iridovirus-encoded RNase III proteins may be in involved in the processing of viral or cellular RNAs, rather than suppression of RNAi.

Viral dsRNA triggers a sequence-specific RNAi response in invertebrates, but may additionally induce a sequence-independent antiviral response in marine shrimp (*Litopenaeus vannamei*) and honey bees (*Apis mellifera*) (60,61). Notably, IIV-6 infects a broad range of invertebrate hosts under natural and experimental conditions, including honey bees and penaeid shrimp (53,62,63). Therefore, it will be interesting to analyze whether 340R (and perhaps 142R) antagonizes putative dsRNA-induced transcriptional responses in invertebrates.

In this study, we used RNAi reporter assays to detect VSR activity for candidate proteins. However, these assays have their limitations, since they may fail to identify cis-acting VSRs (64) and host species-specific VSRs (65). Moreover, these assays could identify VSR activity of dsRNA-binding proteins that are unlikely to suppress RNAi under natural conditions (66). To analyze whether viral dsRNA-binding proteins function as VSRs *in vivo*, it is important to study replication of VSR-defective virus mutants in an RNAi competent host as well as in an RNAi-deficient host. Since no strategies are yet available to genetically manipulate IIV-6, the role of 340R in infection remains to be established. Nevertheless, we demonstrated that RNAi is suppressed in IIV-6-infected cells and that 340R, like other VSRs, rescues replication of FHV ΔB2 (12,13,34,43,47,67–69), suggesting that 340R is a *bona fide* VSR. We and others previously reported that the DNA virus IIV-6 is restricted by an antiviral RNAi response (35,36). Our finding that IIV-6 340R antagonizes RNAi provides further support for an antiviral RNAi response to DNA virus infection in insects.

## SUPPLEMENTARY DATA

Supplementary Data are available at NAR Online.

SUPPLEMENTARY DATA
