# Supplementary material for: A dsRNA-binding protein of a complex invertebrate DNA virus suppresses the Drosophila RNAi response
Source: Nucleic Acids Res. 2014 Oct 1;42(19):12237–48. doi: 10.1093/nar/gku910 (PMC4231766; doi:10.1093/nar/gku910)
Supplement: SUPPLEMENTARY DATA [file supp_42_19_12237__index.html]

A dsRNA-binding protein of a complex invertebrate DNA virus suppresses the Drosophila RNAi response — A dsRNA-binding protein of a complex invertebrate DNA virus suppresses the Drosophila RNAi response — SUPPLEMENTARY DATA 

# A dsRNA-binding protein of a complex invertebrate DNA virus suppresses the *Drosophila* RNAi response

## SUPPLEMENTARY DATA

**Files in this Data Supplement:**

- SUPPLEMENTARY DATA
