# Supplementary material for: A dsRNA-binding protein of a complex invertebrate DNA virus suppresses the Drosophila RNAi response
Source: Nucleic Acids Res. 2014 Oct 1;42(19):12237–48. doi: 10.1093/nar/gku910 (PMC4231766; doi:10.1093/nar/gku910)
Supplement: SUPPLEMENTARY DATA [file supp_gku910_nar-02056-v-2014-File008.pdf]

Supplementary Data

A dsRNA-binding protein of a complex invertebrate DNA virus suppresses the *Drosophila* RNAi response

Alfred W. Bronkhorst[1], Koen W.R. van Cleef[1], Hanka Venselaar[2], and Ronald P. van Rij[1,*]

[1]Department of Medical Microbiology, Radboud University Medical Center, Radboud Institute for Molecular Life Sciences, P.O. Box 9101, 6500 HB Nijmegen, The Netherlands
[2]Center for Molecular and Biomolecular Informatics, Radboud University Medical Center, Radboud Institute for Molecular Life Sciences, P.O. Box 9101, 6500 HB Nijmegen, The Netherlands
[*]To whom correspondence should be addressed. Tel: +31 24 3617574; Email: ronald.vanrij@radboudumc.nl

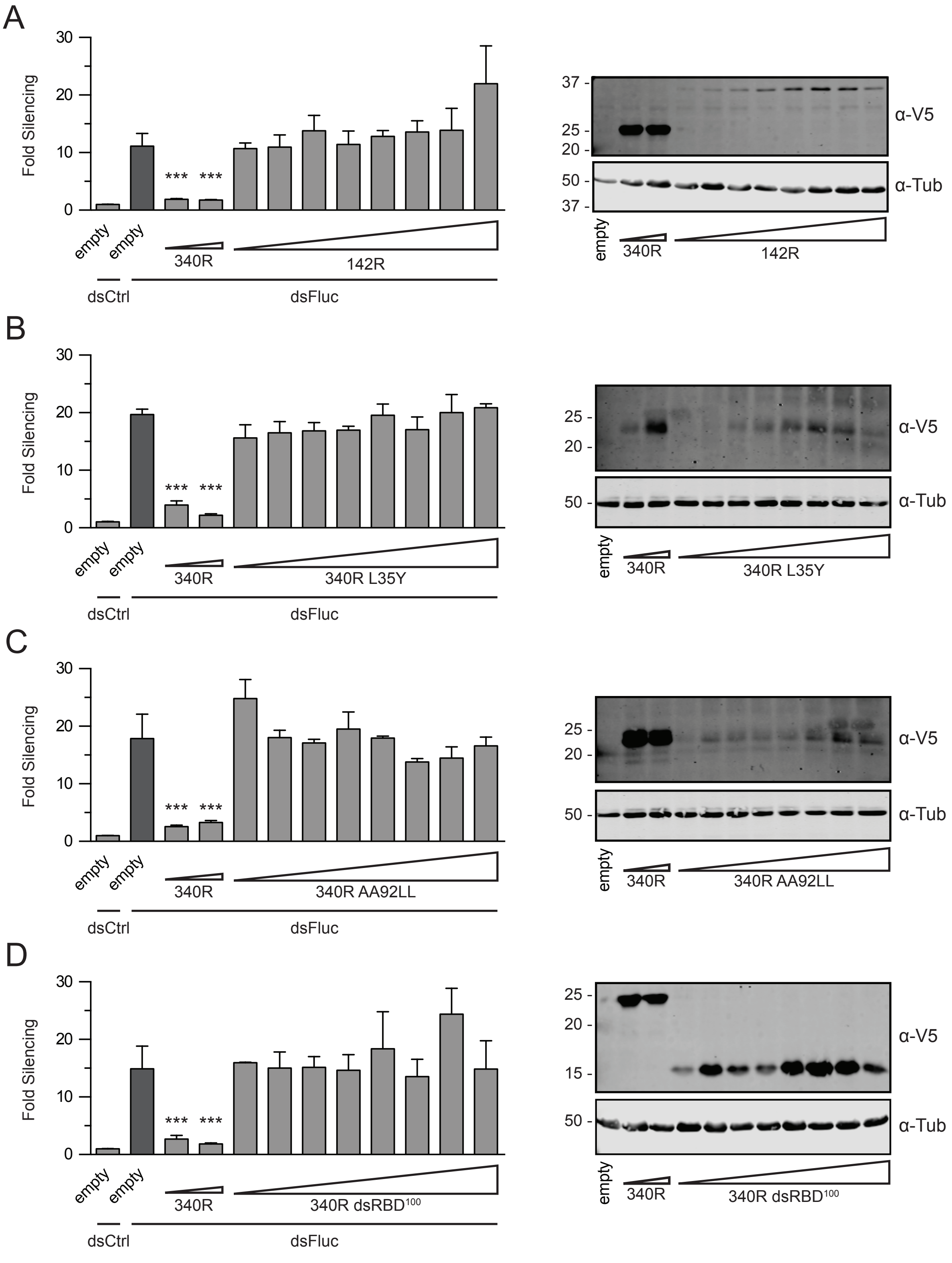

Figure S1. Increasing the expression of 142R, 340R L35Y, 340R AA92LL, and 340R dsRBD^{100} does not result in detectable VSR activity. Left panels, RNAi reporter assays in S2R+ cells transfected with luciferase reporter plasmids and either a control plasmid (empty) or increasing amounts (50 to 400 ng) of expression plasmids encoding 142R (A), the 340R point mutants L35Y (B) and AA92LL (C), or 340R dsRBD^{100} (D). In all panels, WT 340R (25 and 50 ng) was included as a positive control. RNAi was induced by addition of long Fluc dsRNA (dsFluc) to the culture supernatant. Fluc counts were normalized to Rluc counts and expressed as fold silencing relative to control treatment (dsCtrl). Bars in all panels represent the means and standard deviations of three independent samples. Difference in RNAi efficiency compared to controls (dark grey bars) was analyzed by one-way ANOVA followed by a post-hoc Dunnett's test. $^*P \leq 0.05$; $^{**}P \leq 0.01$; $^{***}P \leq 0.001$. Right panels, Western blot analyses of V5 epitope-tagged VSR constructs from cell lysates of the corresponding RNAi reporter assays. Proteins were detected using anti-V5 antibodies (α-V5). A polyclonal anti-α-tubulin (α-Tub) antibody was used as a loading control. Molecular mass (in kDa) is indicated on the left of the image. The predicted molecular weights for 340R WT and the point mutants L35Y and AA92LL are 23 kDa. The predicted molecular weights for 340R dsRBD^{100} and 142R are 14.7 kDa and 37 kDa, respectively.

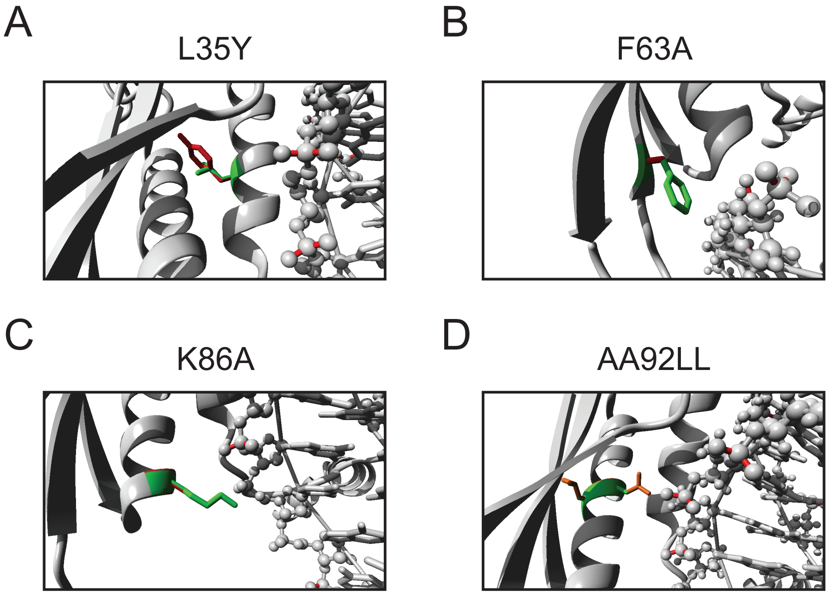

Figure S2. Homology models of WT and mutant 340R in complex with dsRNA. In all panels, the WT residue is shown in green and the mutant residue in red. The RNA is presented in ball-and-stick view with all atoms shown. (A) The conserved residue L35 is located in alpha helix 1 (α1), with its side-chain buried in the core of the protein. Putative RNA interacting residues are located in α1, on the opposite side of L35. The L to Y substitution is likely to disturb the local structure, thereby affecting RNA interactions. (B) The highly conserved residue F63 is surface-exposed, with its side-chain positioned towards the dsRNA, and was proposed to properly position the long flexible dsRNA-interacting Lysine side-chains in Xlrbpa (1). Indeed, mutational analysis of Xlrbpa F145, which corresponds to F63 in 340R, revealed that this residue is required for efficient RNA binding (2). (C) As for K89 (Figure 2B), the side-chain of residue K86 is positioned towards the phosphate backbone of the RNA. The K86A substitution is likely to abolish the interaction between the positively-charged Lysine and the negatively-charged phosphates. (D) The AA92LL substitutions are located in the core of the protein and are expected to severely disturb the local structure and thereby affect the interaction with dsRNA-binding region 3.

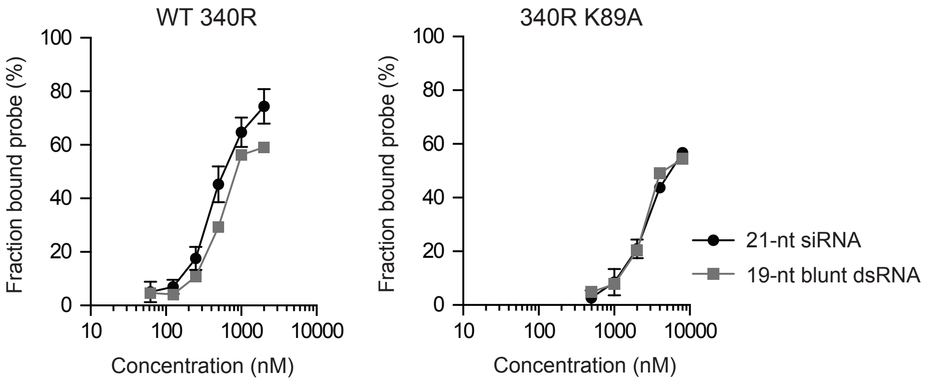

Figure S3. 340R binds 21-nt siRNAs and 19-nt blunt dsRNA with similar affinities. Quantification of the fraction bound 21-nt siRNA (black line) and 19-nt blunt dsRNA (grey line) at different protein concentrations of WT 340R (A) and the K89A mutant (B). Data points for siRNA shifts (black lines) represent means and standard deviations of three independent experiments. Please note that both panels are merged images of figure 3D and 3F.

Table S1: Primers used for cloning and site-directed mutagenesis

**Cloning into pAc5.1-V5-HIS B & site-directed mutagenesis**		
Construct	Forward primer (5'–3')	Reverse primer (5'–3')
142R	AGTTCTAGAAACATGGAAAACAATATTGAAAATTTTGATC	GGTTCTAGATTACATAAAAGCTTTAAACCTGTCTGG
340R-WT	AGTTCTAGAAACATGGAAAAACAAAAAGATAACGTAACTTC	GGTTCTAGATTAAATATCGTCTATATCAACAGAGTCTTC
340R-dsRBD^{100}	AGTTCTAGAAACATGGAAAAACAAAAAGATAACGTAACTTC	GGTTCTAGATAATTCTTTAATAGTTTTAAATGCTGC
340R-K86A	GGTAGCGGTTTATCCATGGCCGAAGCCAAAAAAAAATGC	GCATTTTTTTTTGGCTTCGGCCATGGATAAACCGCTACC
340R-K89A	CATGAAAGAAGCCGCAAAAAAATGCAGC	GCTGCATTTTTTTGCGGCTTCTTTCATG
340R-F63A	GATCATTCTCCAACGGCTAATATTGAATGTCG	CGACATTCAATATTAGCCGTTGGAGAATGATC
340R-AA92LL	CCATGAAAGAAGCCAAAAAAAAATCTCCTCTTTAAAACTAT	ATAGTTTTAAAGAGGAGATTTTTTTTTGGCTTCTTTCATGG
340R-L35Y	CAATTGGATTTTACAACGAATTTTGTC	GACAAAATTCGTTGTAAAATCCAATTG
**Cloning into pMAL-C2X**		
Construct	Forward primer (5'–3')	Reverse primer (5'–3')
DCV 1A	AGTGAATTCATGGAATCTGATAAAAGTATGGCC	GGTGTCGACTTAAACGGGTCCAGGGTTAGTCTC
340R-WT	AGTGAATTCATGGAAAAACAAAAAGATAACGTAACTTC	GGTGTCGACTTAAATATCGTCTATATCAACAGAGTCTTC
340R-K89A	AGTGAATTCATGGAAAAACAAAAAGATAACGTAACTTC	GGTGTCGACTTAAATATCGTCTATATCAACAGAGTCTTC
340R-dsRBD^{100}	AGTGAATTCATGGAAAAACAAAAAGATAACGTAACTTC	GGTGTCGACTTATAATTCTTTAATAGTTTTAAATGCTGC

Introduced restriction sites of XbaI, EcoRI and SalI are underlined.

Supplementary References

1. Ryter, J.M. and Schultz, S.C. (1998) Molecular basis of double-stranded RNA-protein interactions: structure of a dsRNA-binding domain complexed with dsRNA. *Embo J*, **17**, 7505-7513.
2. Krovat, B.C. and Jantsch, M.F. (1996) Comparative mutational analysis of the double-stranded RNA binding domains of Xenopus laevis RNA-binding protein A. *J Biol Chem*, **271**, 28112-28119.
